# Effects of Pulsed-Wave Chromotherapy and Guided Relaxation on the Theta-Alpha Oscillation During Arrest Reaction

**DOI:** 10.3389/fpsyg.2022.792872

**Published:** 2022-03-03

**Authors:** Guy Cheron, Dominique Ristori, Mathieu Petieau, Cédric Simar, David Zarka, Ana-Maria Cebolla

**Affiliations:** ^1^Laboratory of Neurophysiology and Movement Biomechanics, Université Libre de Bruxelles, Brussels, Belgium; ^2^ULB Neuroscience Institute, Université Libre de Bruxelles, Brussels, Belgium; ^3^Laboratory of Neuroscience, Université de Mons, Mons, Belgium; ^4^Machine Learning Group, Computer Science Department, Université Libre de Bruxelles, Brussels, Belgium

**Keywords:** chromotherapy, EEG, visual evoked potentials, alpha-theta oscillations, wellness

## Abstract

The search for the best wellness practice has promoted the development of devices integrating different technologies and guided meditation. However, the final effects on the electrical activity of the brain remain relatively sparse. Here, we have analyzed of the alpha and theta electroencephalographic oscillations during the realization of the arrest reaction (AR; eyes close/eyes open transition) when a chromotherapy session performed in a dedicated room [*Rebalance* (*RB*) device], with an ergonomic bed integrating pulsed-wave light (PWL) stimulation, guided breathing, and body scan exercises. We demonstrated that the PWL induced an evoked-related potential characterized by the N2-P3 components maximally recorded on the fronto-central areas and accompanied by an event-related synchronization (ERS) of the delta–theta–alpha oscillations. The power of the alpha and theta oscillations was analyzed during repeated ARs testing realized along with the whole *RB* session. We showed that the power of the alpha and theta oscillations was significantly increased during the session in comparison to their values recorded before. Of the 14 participants, 11 and 6 showed a significant power increase of the alpha and theta oscillations, respectively. These increased powers were not observed in two different control groups (*n* = 28) who stayed passively outside or inside the *RB* room but without any type of stimulation. These preliminary results suggest that PWL chromotherapy and guided relaxation induce measurable electrical brain changes that could be beneficial under neuropsychiatric perspectives.

## Introduction

The development of different types of physical and mental exercises, such as relaxation, guided breathing (Doll et al., 2016), mental imagery (Cebolla et al., 2017), and meditation (Zorn et al., 2021), has acquired a paramount position in the Western world because of the increasing popular research of a singular serenity sustained by wellness practice (Cahn and Polich, 2006; Ryznar and Levine, 2021).

As the mindfulness-based stress reduction method proposed by Kabat-Zinn (1982), the scientific interest for mindfulness intervention has augmented exponentially (Creswell, 2017). Differently operationalized, mindfulness intervention consists of helping subjects to pay attention to their ongoing moment experience and to reach a self-regulated attentional state, emphasizing curiosity and openness, and relying on attention stability and meta-awareness (Quaglia et al., 2015, 2019). The effects of meditation practice on morphological plasticity of the brain have been reviewed by the meta-analysis of Fox et al. (2014), concluding its existence in meditators of consistent and medium-sized brain structure differences in the gray and white matter of the prefrontal cortex, sensory cortices, insula, and hippocampus. In parallel, EEG studies on the meditation effects have demonstrated the potentiation of the theta and alpha oscillations during these practices (Aftanas and Golocheikine, 2001; Lagopoulos et al., 2009; Henz and Schöllhorn, 2017).

Because of the positive outcomes of mindfulness practice on stress (Gotink et al., 2016), chronic pain (Hilton et al., 2017; Poletti et al., 2021), obesity (Lattimore, 2020), sleep (Kwekkeboom and Bratzke, 2016), emotion (Lutz et al., 2016; Kral et al., 2018; Makowski et al., 2019), and cognition (Tang et al., 2015), the number of practitioners has significantly increased in recent years. However, it is well known that the practice of meditation and even more mindfulness is not given to everyone. This is one reason environments like chromotherapy rooms (Azeemi and Raza, 2005; Azeemi et al., 2019) suitable for meditation, such as the present *Rebalance* (*RB*) device, have been developed. In this technological setup, the search for optimal comfort was carried out within an enclosure whose ceiling includes a continuous series of LEDs arranged in bars radiating from the center. The successive lighting of these LEDs produces a visual sensation of waves in movement that we have qualified for pulsed-wave light (PWL) stimulation. These eccentric and concentric visual stimulations at the theta–alpha rhythm may induce oscillatory entrainment. This brainwave entrainment is a new non-invasive technique able to reconfigure the oscillatory dynamics of the brain for a better mental health and cognitive performance (Lakatos et al., 2019; Addante et al., 2021; Poltorak, 2021; Quirk et al., 2021; Yadav et al., 2021).

In this context, the two main types of EEG oscillation, theta and alpha, represent appropriate rhythmic activities to be investigated during an *RB* session. The cortical theta is mainly influenced by the hippocampal theta oscillation, which is in turn modulated by the medial septum-diagonal band of Broca and the supramamillary nucleus playing the role of a subcortical theta pacemaker (Vertes and Kocsis, 1997; Vertes et al., 2004). A 6-Hz theta peak has been recorded in the human hippocampus (Kahana et al., 1999). A “slow-theta” (2.5–5 Hz) related to successful associative memory (Pastötter and Bäuml, 2014; Lega et al., 2016) and a “fast-theta” (5–9 Hz) related to phase reset and reinstatement of oscillatory patterns for successful recollection were well defined (Kota et al., 2020; Ter Wal et al., 2021).

Likewise, for the theta rhythm, the cortical alpha is influenced by a complex interplay between cortical and subcortical generators. Considered as the major alpha pacemaker, the thalamus (Lorincz et al., 2009) is additionally modulated by the reticular formation and the pulvinar nucleus (Saalmann and Kastner, 2009, 2011). Peaking at about 10 Hz, the alpha rhythm dominates the EEG and governs different sensorimotor modalities. It is thus well suited for studying the effect of the mixing of the different stimulations offered by the *RB* device. In addition, the alpha oscillation is considered as a global rhythmic coordinator of the brain activity providing top-down signals from higher hierarchical cortical areas to lower cortical and subcortical regions (e.g., thalamus and pulvinar) (Halgren et al., 2019). In this way, fluctuations in the top-down modulated alpha oscillation influence how visual information is treated during attention processes (Sauseng et al., 2005; Kelly et al., 2006).

The combination of different wellness practices (Zgierska et al., 2016; Hanley et al., 2021; Liu et al., 2021; Polaski et al., 2021) has demonstrated an increased efficiency promoting the development of devices, such as chromotherapy room. Chromotherapy is an ancient alternative medicine, often considered as a pseudoscience, planning to use the energy of electromagnetic radiations in the visible light spectrum to produce physiological and or psychological effects in humans.

Different commercially available systems have been developed for facilitating wellness. It has been advanced that the entrance into such chromotherapy rooms facilitated mindfulness and related practice. In addition, the exposition to oscillating red, green, and blue lights produced significant effects on the cardio-autonomic control by increasing the heart rate variability (Grote et al., 2013). By using the similar alternation of red, green, and blue light at 10 s corresponding to the blood pressure oscillation (as used in the present RB device), a resonance effect between the internal blood pressure rhythms and the external light oscillation may induce positive effects on cardio-autonomic regulation and allow subjective wellbeing (Grote et al., 2013). In the same vein, Tallon-Baudry et al. (2018) suggest that the conscious perception of visual input is the consequence of the integration of the visual content with the neural monitoring of bodily state in which the visceral inputs as the heart rate signaling are the important elements (Park et al., 2014, 2016). In this context, it was shown that the alpha peak frequency was correlated with the heart rate during wakefulness (Lechinger et al., 2015), and the alpha power was higher when attention was directed to the heart instead of a visual task (Villena-González et al., 2017).

The motivation to use repeated transition from the eye-closed (EC) to the eye-opened (EO), called the arrest reaction (AR) by Berger (1929) was 2-fold: (1) it is a convenient and reproducible maneuver for the quantification of the dynamics of the alpha rhythm (8–13 Hz), which is higher during the EC than during the EO condition (Cheron et al., 2006); (2) it follows the practice of mindfulness, which often requires the closing of the eyes to facilitate both exteroceptions- and interoceptions, (Jao et al., 2013; Bastarrika-Iriarte and Caballero-Gaudes, 2019). This EC state is often used as a physiological baseline state for the default mode network (DMN) of the human brain, upon which the performance of goal-directed behaviors can be realized (Raichle et al., 2001).

Based on the main characteristic of the RB device offering a combination of (1) meditation facilities (guided breathing and body scan), (2) chromotherapy, and (3) PWL entrainment, we hypothesize that the RB sessions can induce a power increase of the alpha and theta oscillations recorded at rest in the EC and EO state. For this, after studying the effect of PWL on the EEG dynamics, we have quantified the power spectrum of the alpha and theta EEG oscillations during repeated AR tests performed after each of the *RB* sessions.

## Materials and Methods

### Participants

The theta–alpha oscillation power spectrum variations were analyzed during AR in a total of 42 participants. These were separated into three age-matched groups (ranging from 22 to 65 years); the action in the *RB* (AIR) group (testing of the *RB* effect) (*n* = 14, female *n* = 5, male *n* = 9) and two control groups, the passive in the *RB* (PIR) group staying passively in the *RB* bed [*n* = 10, (female *n* = 4, male *n* = 6)] and the passive outside *RB* (POR) where participants were comfortably seated outside of the *RB* bed (*n* = 18, female *n* = 10, male *n* = 8). All participants were right-handed and had no neurological condition. The two control groups (non-athletes) were studied to verify the absence of modifications in the theta–alpha power along with the same experimental procedure but without the *RB* stimulation.

The effects of the combined stimulation (chromotherapy PWL + breathing and body scan exercises) provided by the *RB* system on the EEG oscillations were collected in the AIR group. Among the 14 participants, 10 of them were elite Belgian athletes, the older participant was a former elite athlete and present coach. The four remaining participants were non-elite athletes. Three of these participants have already experienced the *RB* system 1 month before the present study. In the PIR control group, participants have no previous practice of the *RB* system and were asked to lie in the *RB* bed and to stay calm, to keep their eyes open, and just to follow the audio orders. In the POR control group, the participants were asked to stay relaxed comfortably (Supplementary Table 1) and to eat, with their eyes continuously closed while their EEG was recorded for 34 min.

Each participant gave informed consent to the experimental procedures. All experimental protocols were approved by the Ethic comity of Université Libre de Bruxelles, CHU Brugmann, and were conducted in conformity with the European Union directive 2001/20/EC of the European Parliament.

### The Rebalance Device

**FIGURE 1:**
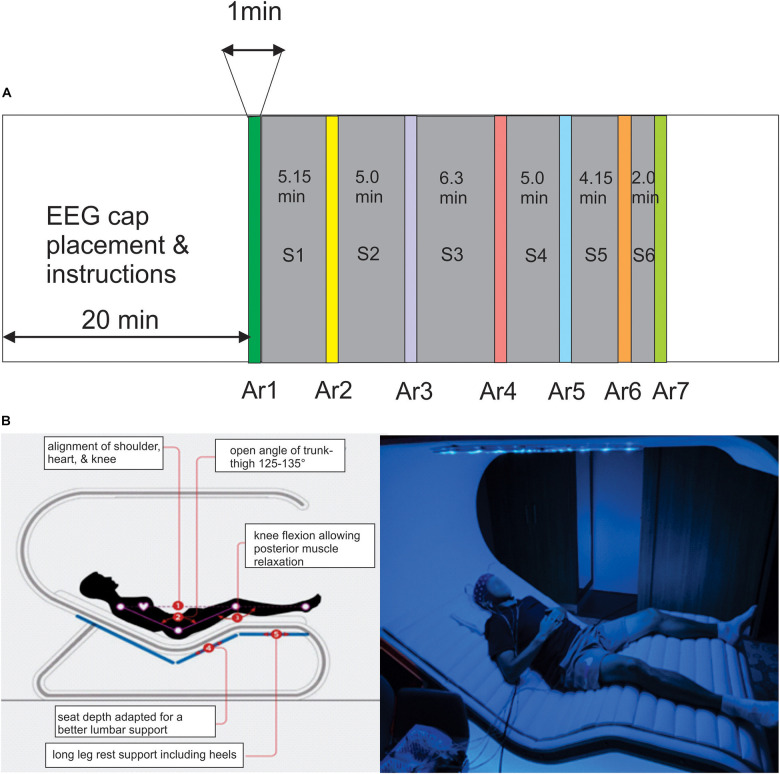
**(A)** The timing of the Rebalance (RB) session consisted of the succession of six modules interrupted by the arrest reaction (AR) testing (AR2–AR7) during which the power of the alpha oscillation was measured, AR1 is the control testing realized before the session. **(B)** Illustration of the RB impulse system in which the participants are comfortably placed in supine position and equipped with the EEG cap recording and earphones.

This device consists of an ergonomic bed integrating chromotherapy system (Figure 1B^1^, France). The covering top includes a circle of 16 radial arms each one including an array of LEDs providing a continuous or intermittent rhythmic wave (pulsed-wave light, PWL) presenting 8 different color lightings ranging from purple (415 nm) to red (720 nm) with different levels of illuminance ranging from 7 to 120 lx. The whole *RB* session consisted of 27.6 min during which chromotherapy stimulations and breathing exercises are proposed in six successive sub-sessions (S1–S6) (Figure 1A and Supplementary Table 1). The breathing exercises are voice-guided and composed of a cycle of five counts for inspiration, five counts for keeping the air at the end of inspiration followed by five counts for expiration, and five counts for keeping the expiration volume before reinitiating the cycle. These breathing exercises only occurred during S1, S3, and S4. A guided body scan exercise was performed only during S2.

### EEG Data Treatment

#### Cleaning of the Data

EEG data were recorded with a POR (Supplementary Table 1) system eego™ sports (EEG system LE-200, ANT Neuro b.v., Enschede, Netherlands) comprising an active-shield cap comfortably adjusted to the participant’s head using 64 Ag/AgCl sintered ring electrodes, the shielded co-axial cables (WaveGuard™ original cap; 10/10 electrode system placements) connected to an amplifier (ANT DC-eego amplifiers 2 kHz; CE Class IIa medical device), and a tablet [TRAVELline T10-B5 Pro Tablet, Atom (TM) x5-Z8350 1.44 GHz] placed close to the participant. The signals were recorded and displayed online *via* a Wi-Fi connection with an off-field computer (Dell Inc., Intel(R) Core™ i7-10510U). All recordings were initially referenced to the frontal CPz electrode. Vertical and horizontal eye movements (EOG) were recorded bipolarly. All electrode impedances were maintained below 5 kΩ. Scalp potentials were amplified and digitized at a rate of 2.048 Hz using a resolution of 16 bit (range 11 mV). To correct the dampening effect of the oscillation amplitude close to the reference point, we transformed the data by subtracting the average activity across all electrodes (i.e., average referencing). After downsampling to 512 Hz, a zero-phase IIR band-pass filter from 1 to 40 Hz was applied. The artifactual portions of the EEG data were rejected after an appropriate independent component analysis (ICA) performed with the EEGLAB software (v2021.0) (Delorme and Makeig, 2004).

#### Event-Related Potentials

For the time domain analysis, the event-related potentials (ERPs) were calculated by averaging baseline-corrected epochs extracted from − 1 to 1 s around the onset of the PWL stimulation occurring at a variable frequency of 3.4–5.6–8.2–10.8 Hz during the 6 *RB stimulation* sessions (S1–S6). A total of 214 trials per subject, including these different PWLs, were used for averaging. A pulse was produced by the system generating the PWL at the onset of each stimulation and that without any jitter. The ERP and the event-related spectral perturbation (ERSP) were thus triggered by these external pulses precisely synchronized with the onset of the PWL stimulation. This grand average analysis was only performed on seven participants’ data for which the electrical artifacts due to the PWL were easily identified and deleted by the ICA procedure and for which EMG and movement artifacts were easily removed. We used the scalp topography, temporal activity localization, and spectral magnitude criteria to identify the ICA related to artifacts (a maximum of four ICA components per participant were deleted).

#### Event-Related Spectral Perturbation and Intertrial Coherency

For the time-frequency analysis of EEG oscillations, we first calculated the baseline-normalized ERSP (Pfurtscheller and Neuper, 1994; Makeig et al., 2002). ERSP measures variations in the power spectrum of ongoing rhythms at specific periods and frequency ranges. In ERSP measurements, event-related desynchronization (ERD) indicates a power spectrum reduction while event-related synchronization (ERS) indicates a power spectrum increase. In parallel, the phase-locking of the ongoing oscillations was calculated by the intertrial coherency (ITC) analysis. FFT was used as a spectral decomposition technique from 2 to 40 Hz frequency range and performed on 2,048 time points with a time window of 200 points. A divisive baseline ranging from −1 s to 0 s was used.

#### Power and Frequency Peak Analysis of the Alpha and Theta Oscillation During the Arrest Reaction Testing

**FIGURE 2:**
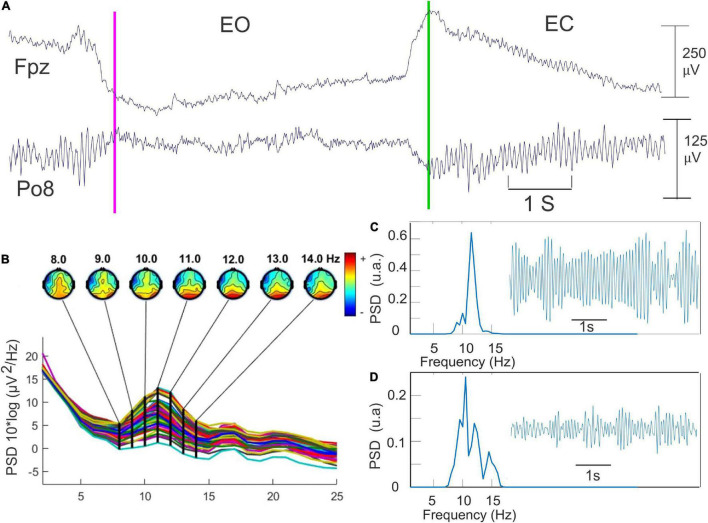
The methodology used for the quantification of the alpha oscillation. **(A)** Detection of opening (pink line) and closing of the eyes (green line) in Fpz recording. The topographical map provided by the EEGLab toolbox for detecting the scalp electrode presenting the higher alpha power. **(B)** An example of 6 s of the filtered EEG data (8–14 Hz) recorded during the eye-closed (EC) state. **(C)** Fast Fourier transform of this data set corresponding of the EC state. **(D)** the same procedure as in C but during the EO state.

The occurrence of the EEG signals corresponding to the eyes opening and eyes closing was initially detected by visual inspection of the raw signals of the frontal electrodes (Figure 2A). The AR testing was performed before (AR1), during (AR2–AR6), and after (AR7) the whole *RB* session. Given the time constraints linked to the optimal use of the *RB* stimulations, each AR testing consisted of only five repetitions of 6 s eyes-closed and 6 s eyes-opened representing 1 min of recording. The duration of the six modules of *RB* stimulation ranged from 2 to 6.3 min. The total duration of the *RB* session was 34.6 min (27.6 min of stimulation and 7 min for the AR testing). About 20 min was needed for the placement of the EEG cap (Figure 2). The quantification of the alpha oscillation was repeatedly performed on the filtered signals (8–14 Hz) of each of these 6 s periods recorded by the parieto-occipital electrodes showing the bigger alpha amplitude (Figures 2B,C). We standardized the amplitude of these alpha oscillations by subtracting the mean amplitude from the original signals and dividing the result by the SD. The power spectral density (PSD) was then calculated using Welch’s averaged periodograms with a one-second Hamming window and a frequency resolution of 0.45 Hz with an overlapping of 50%. The maximal power and the frequency of the alpha were automatically extracted from the PSD data.

The individual frequency peak of the alpha oscillation has been used as a biomarker of stress and arousal during sports exercise but also as a stable neurophysiological marker (Christie et al., 2017) and a useful measurement for the evaluation of stress and fatigue recovery (Bertollo et al., 2017).

The same procedure was applied for the analysis of low and high theta oscillations. In this case, the EC and EO periods were pooled together, and appropriate filtering was used, 3.0–5.5 Hz and 5.5–8 Hz for the low and the high theta oscillations, respectively. The theta power quantifications were performed on the Cz electrode, which showed a greater amplitude and may thus correspond to the frontal midline theta (FM Theta), which is known to be extended from the anterior part of the frontal to the centro-parietal cortex (Piarulli et al., 2018).

In addition to the power analysis, the individual alpha and theta (low and high) peak frequencies (iPF) were measured during different AR testing methods (AR1–AR7).

### Statistical Analysis

The independent variables are the different *RB* sessions, the alpha and theta powers while the dependent variables are the related frequency peaks measured during AR1–AR7. As these latter variables could be influenced by the *RB* active session, the *RB* environment by itself (without any stimulation), and the time-lapse, we also included two control groups one being inside the passive *RB* system (the PIR group) and the other one being outside the *RB* system (the POR group). Statistical analysis within each subject was followed by group statistics. For each subject of the AIR group, we tested the null hypothesis that the alpha and theta powers measured during the different AR periods (AR2–AR7) were not modified by the *RB* session (AR1 = AR2, AR1 = AR3, AR1 = AR4, AR1 = AR5, AR1 = AR6, AR1 = AR7). For this, the homogeneity and normality of the variances were first checked by the Bartlett and Shapiro–Wilk test. Then, parametric (*t*-test and ANOVA) or non-parametric (Kruskal–Wallis test) and the *nparcomp* function (Konietschke et al., 2015) were applied for the comparative analysis between the AR1 (control) and AR2–AR7 (*RB*) using the software R version 4.0.5. The same procedure was applied for the group statistics.

The results are reported as mean ± SD and illustrated in box plots. The level of significance was set at *p* < 0.05. Off-line data treatment and statistics were performed using MATLAB^®^ (R2021a; MathWorks, Natick, MA, United States).

## Results

### Analysis of the Event-Related Potential and Event-Related Spectral Perturbation Produced by the Pulsed-Wave Light

**FIGURE 3:**
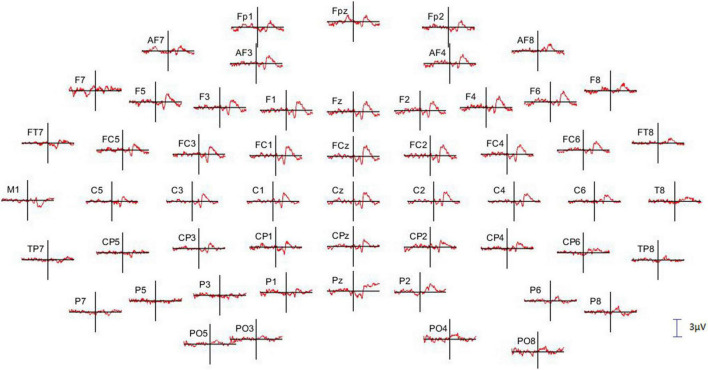
Full scalp array of grand average event-related potential (ERP; *n* = 6 subjects) showing the fronto-central distribution of the N331-P476 components triggered by the onset of the PWL. The vertical lines indicate the onset of the PWL. Only 55 of the 64 electrodes are illustrated, and the missing ones present too many artifacts. The calibration bar corresponds to 3 μV, positivity up.

**FIGURE 4:**
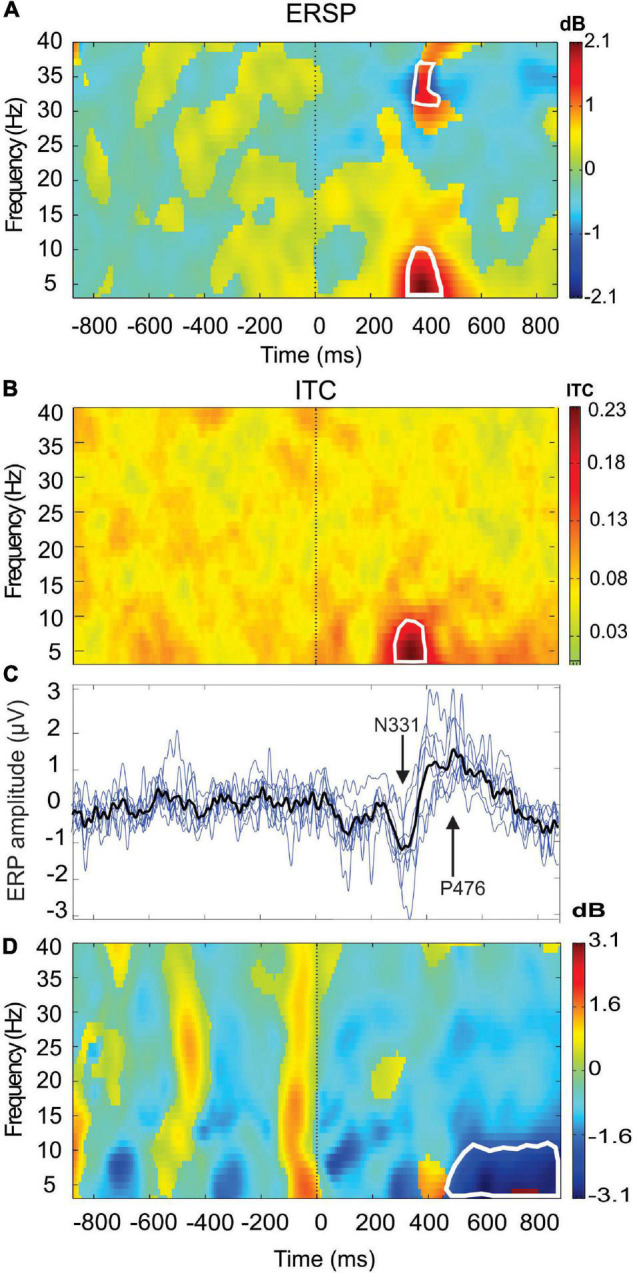
Event-related spectral perturbation (ERSP) **(A)** and intertrial coherency (ITC) **(B)** (grand average *n* = 6 subjects) are recorded at the Fz electrode showing the event-related synchronization (ERS) in the delta-theta–alpha frequency bands concomitant of a theta phase-locking (red area). Also note the presence of beta-gamma ERS. In **(C)**, superimposition of the ERPs of the six single subjects (blue lines) (*n* = 214 PWL stimulation) and the grand average ERP (black line, *n* = 1,284). In **(D)**, ERSP is recorded in PO8 electrodes showing a theta–alpha event-related desynchronization (ERD) (blue area). The areas of statistical significance at *p* < 0.01 are marked by white squares.

Before undertaking the quantification of the alpha oscillation induced by the *RB* session during the AR testing, we have first examined the effect produced by the PWL stimulation on the EEG dynamics. For this, 214 PWLs per subject were used for the ERP and ERSP analysis. Figure 3 illustrated the ERP configuration on the whole scalp showing that the most significant ERP occurred in the fronto-central region with a first negative peaking at 331.6 ± 48.7 ms with an amplitude of 1.60 ± 0.40 μV (*n* = 6) followed by a positive component peaking at 476.0 ± 58.7 ms with an amplitude of 1.68 ± 0.68 μV (*n* = 6). These N331-P476 components were accompanied by an ERS of the delta–theta–alpha frequency band peaking at the latency of 354.4 ± 69.1 ms and an ITC in the same frequency band of 20–40% of the trials occurring at the transition between the N331 and the P476 component (Figures 4A–C). Despite the absence of a reliable ERP in the parieto-occipital region (Figures 3, 4D), the ERSP analysis showed the presence of long duration (from 200 to 1,000 ms) delta-theta–alpha ERD (Figure 4D). Even though our working hypothesis was focused on the alpha oscillation, the presence of a significant ERS in the theta band induced by the PWL stimulation has motivated the analysis of the power of the low and high theta oscillation during the AR testing.

### Analysis of the Alpha Power During the Arrest Reaction Testing

For each subject, the alpha analysis performed on the EEG data filtered from 8 to 13 Hz was therefore carried out 35 times during the 34.6 min of the *RB* session distributed according to the six different types of PWL stimulation and breathing exercises.

For the majority of participants (11 of 14), a significant increase in the alpha oscillation between at least the AR1 and one of the other AR tests (AR2–AR7) was reported in the E0 (*n* = 8 participants) and the EC (*n* = 5 participants). Two participants increased the alpha power in both EO and EC conditions, and in three participants the alpha power was not significantly modified by the *RB* sessions.

**FIGURE 5:**
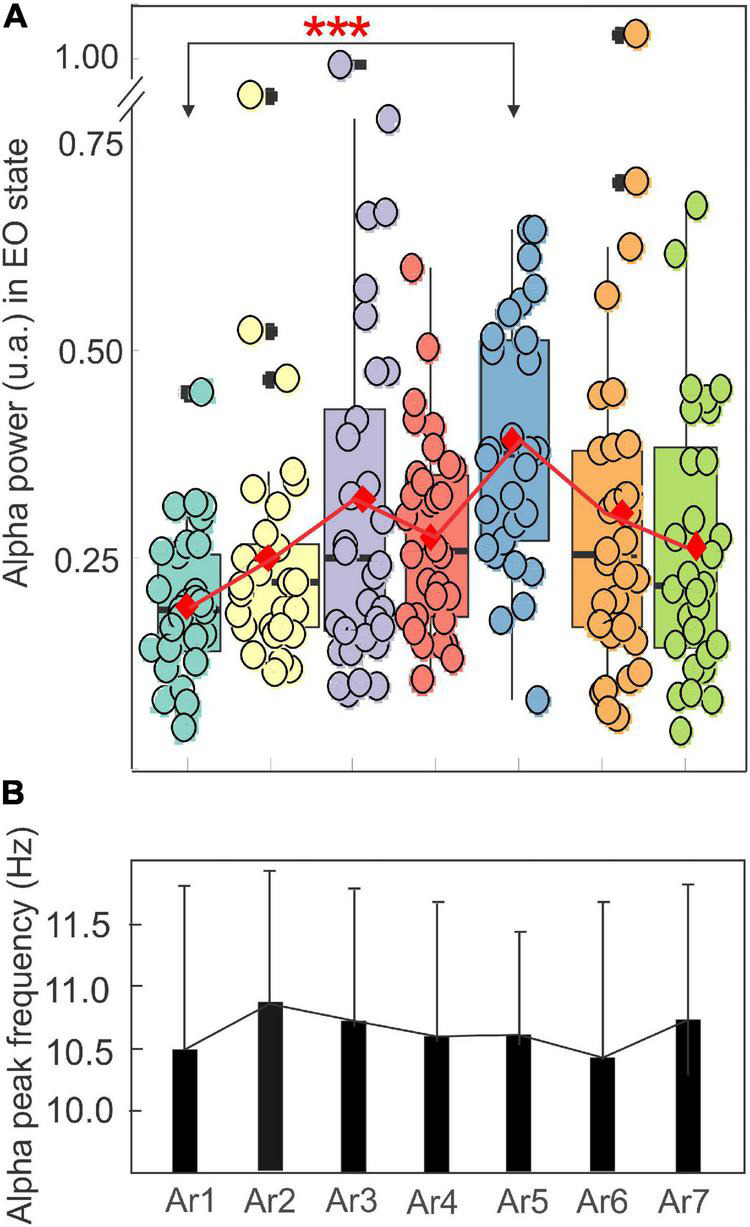
Evolution of the mean ± SD of the power in a box plot configuration **(A)** and of the frequency peak **(B)** of the alpha rhythm with the eyes open from AR1 (control before the RB session) to AR7, in the eight subjects who showed an increase of the alpha oscillation for at least one AR testing (AR2–AR7).

Figure 5A illustrates the changes in the spectral power of the alpha rhythm measured in the EO state between all *RB* sessions (S1–S6) in the eight subjects who showed a significant increase during at least one of the six AR tests (AR2–AR7). In the control situation (AR1), i.e., before the session, the mean power of the alpha was 0.19 ± 0.08 and a significant increase was observed in AR5 for which a value of 0.39 ± 0.15 was reached (*p* = 0.00002, Kruskal–Wallis test) after the session S4. It is interesting to note that the S4 session consisted of the Generic Square breathing maneuver in addition to PWL stimulation. For the other measurements (AR2, AR3, AR4, AR6, and AR7), the observed increases were not statistically significant and a break in the progressive increase from AR1 to AR5 was observed. When these alpha power values were normalized, they reached 224% and 144% during AR5 and AR4, respectively, of the control value (*p* < 0.00007 and *p* < 0.0009, Kruskal–Wallis test). The stability of the alpha rhythm individual frequency peak (ranging from 10.48 ± 1.18 to 10.88 ± 1.03 Hz) throughout the *RB* sessions was observed for this group of participants (Figure 5B). When all the participants (*n* = 14) (responders and non-responders) were taken into account, the power increase of the alpha oscillation between AR1 and AR5 in the EO state remained significant (*p* < 0.0009, Kruskal–Wallis test) reaching 169% of the control value.

**FIGURE 6:**
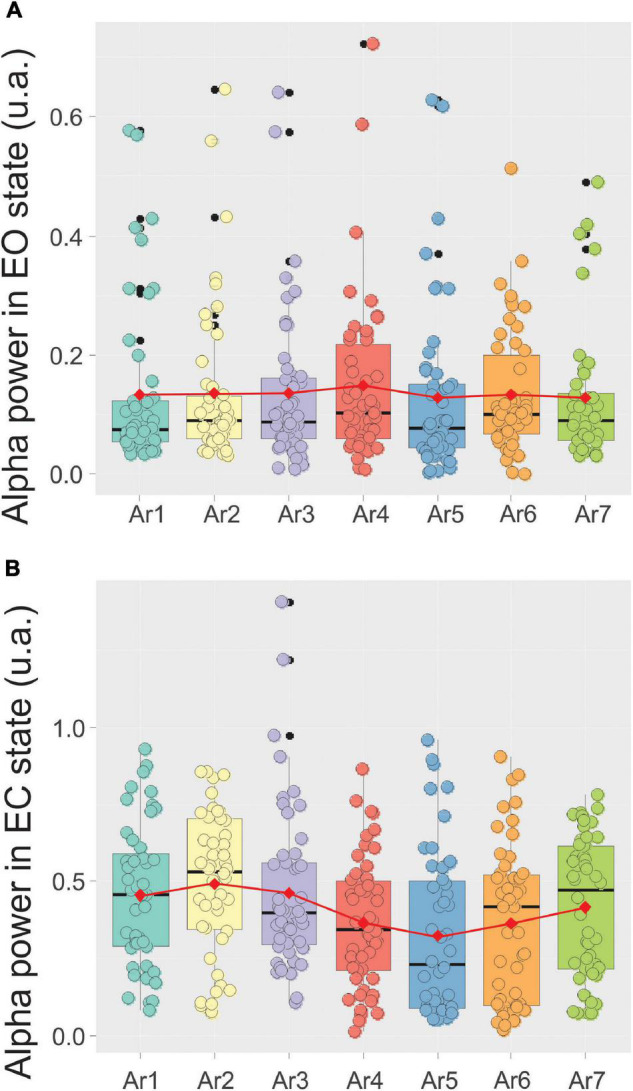
Evolution of the mean ± SD of the alpha power in a box plot configuration in the EC state **(A)** and the eye opened (EO) state **(B)** from AR1 (control before the RB session) to AR7, in the 10 subjects of the first control group.

By contrast, the alpha power remained constant throughout the control situation performed by the first control group in which the participants stay passively in the *RB* system without any type of stimulation. In this situation, the alpha power was 0.13 ± 0.13 before the session (AR1) and never significantly increased higher than 0.14 ± 0.13 (AR4) (Figure 6A).

**FIGURE 7:**
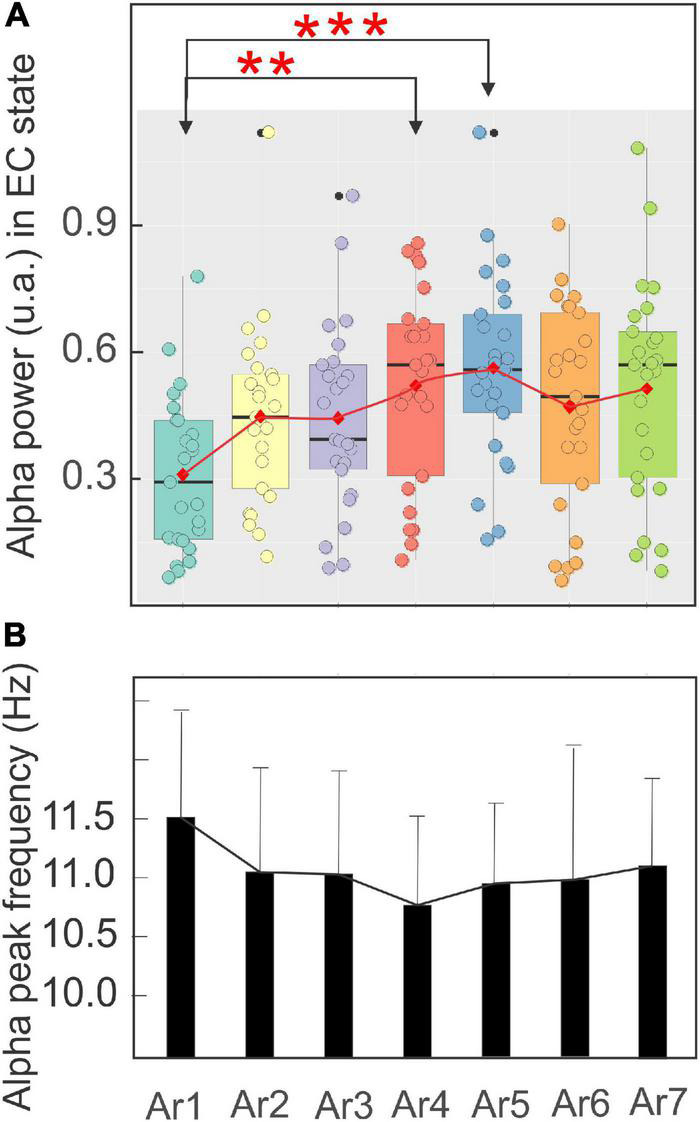
Evolution of the mean ± SD of the power in a boxplot configuration **(A)** and of the frequency peak **(B)** of the alpha rhythm with the eyes closed from AR1 (control before the RB session) to AR7, in the five subjects who showed an increase of the alpha oscillation for at least one AR testing (AR2–AR7). The asterisk indicated the level of significance *p < 0.05 and **p < 0.005.

Figure 7 illustrates, using the same arrangement as in Figure 5, the evolution of the spectral power of the alpha rhythm measured in the EC state between all *RB* sessions in the five subjects exhibiting a significant increase during at least one of the six AR tests. The stability of the alpha rhythm frequency peak for this group of participants (ranging from 10.79 ± 0.73 to 11.51 ± 0.9 Hz) was also observed throughout the *RB* session (Figure 7B). In the control situation (AR1), the mean alpha power was 0.31 ± 0.18 and a significant increase was observed during AR4 where it reached a value of 0.52 ± 0.23 (*p* < 0.03, Kruskal–Wallis test) and maintained a value of 0.56 ± 0.22 (*p* < 0.005) during the AR5 test. For the other measurements (AR2, AR3, AR6, and AR7), the observed increases were not statistically significant. When these alpha power values were normalized, they reached a percentage higher than 152% in each AR testing with the highest score in AR5 of 219%. When all the participants were taken into account, a value of 139% was obtained in AR5 but did not reach a significant level. By contrast, the alpha power recorded in the EC state in the first group of control remained unchanged and was 0.45 ± 0.23 before the session (AR1) and never increased to more than 0.49 ± 0.23 recorded in AR2 (Figure 6B). The stability of the alpha power in the EC state along with a comparable period than the *RB* session was checked in the second group of control for which a value of 0.62 ± 0.05 was obtained before the session (AR1) and never increased to more than 0.63 ± 0.05 during the 34 min of recording.

The analysis of the difference between the alpha power peaks measured during the EC and EO state showed that in 10 participants (of the 14) this value increased during the active *RB* session for at least one AR but reached a statistical significance for only three participants. For the other three participants, this value decreased (with no significance) and in one participant the difference remained unchanged during the *RB* session.

**FIGURE 8:**
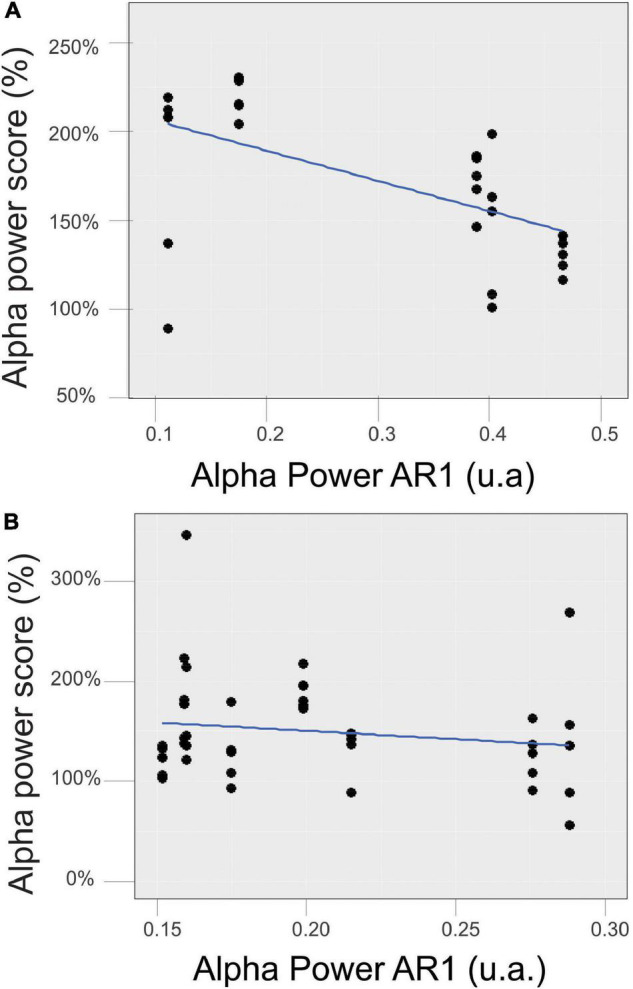
Relationship between the mean initial control value of the alpha power in the EC **(A)** and EO **(B)** states measured during AR1 and the mean score reached during the RB session (AR2–AR7).

We also analyzed the relation between the mean initial control value (AR1) and the mean alpha score reached during the *RB* session (AR2–AR7). We obtained a significant correlation for the EC state (Figure 8A) (*r* = − 0.59, *p* = 0.0005) but not for the EO state (Figure 8B).

### Analysis of the Low Theta and High Theta Power During the Arrest Reaction Testing

**FIGURE 9:**
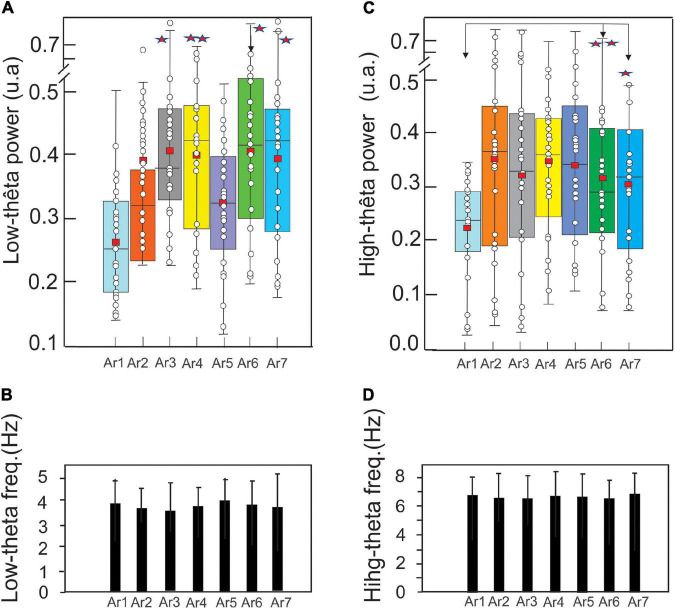
Evolution of the mean ± SD of the low theta **(A)** and high theta **(C)** (EC and EO pooled) power in a boxplot configuration and of the respective frequency peaks **(B,D)** (control before the RB session) to AR7, in the six subjects who showed an increase of these theta oscillations for at least one AR testing (AR2–AR7). The asterisk indicated the level of significance *p < 0.05 and **p < 0.005.

The same analysis procedure was performed for the low-frequency (3–5.5 Hz) and high-frequency (5.5–8 Hz) theta rhythm. Figure 9A illustrates, according to the same arrangement as in Figure 5, the evolution of the spectral power of the low-frequency theta rhythm (EC and EO pooled) during the *RB* session in six subjects exhibiting a significant increase of these rhythms during at least one of the six AR testing.

In the control situation (AR1), the mean power of the low theta was 0.27 ± 0.07 and a significant increase is observed from AR3 where it reached a value of 0.39 ± 0.11 (*p* < 0.001, Kruskal–Wallis test) and 0.41 ± 0.11 (*p* < 0.0001) during the session AR4, 0.40 ± 0.10 during AR6 (*p* < 0.0007) and 0.39 ± 0.12 during AR7 (*p* < 0.0015) (Figure 9A). Expressed in percentage, these increased powers were about 50%. For the other measurements (AR2 and AR5), the observed increases were not statistically significant. Figure 9B illustrates the frequency stability of the low theta rhythm (ranging from 3.67 ± 0.97 to 4.04 ± 0.98 Hz) without a significant variation throughout the *RB* session.

In AR1, the mean power of the high theta was 0.22 ± 0.09 and a significant increase is observed only from AR6 where it is 0.31 ± 0.13 (*p* < 0.002, Kruskal–Wallis test) and maintaining a value of 0.30 ± 0.13 (*p* < 0.02) during the AR7 test. It should be noted that despite a larger average value from AR2 (0.35 ± 0.19), the statistical threshold was not reached because the dispersion of the data was too excessive (Figure 9C). Figure 9D illustrates the stability of the frequency of the high theta rhythm (ranging from 6.54 ± 1.2 to 6.82 ± 1.5 Hz) without a significant variation throughout the *RB* session.

## Discussion

### Event-Related Potential During the Pulsed-Wave Light Stimulation

Our findings first demonstrated that the PWL stimulation *RB* was able to produce an ERP in the fronto-central region characterized by an N331-P476 component accompanied by an ERS of the delta–theta–alpha frequency band. These ERP components can be viewed as the N2-P3 commonly described in the literature (Greimel et al., 2013; Decroix et al., 2020) relative to the involvement of the visual dorsal pathway considered as the “where” pathway originating in the magnocellular layer of the lateral geniculate nucleus (LGN) to the primary visual cortex (V1), forward to the middle temporal area (MT/V5) and then to the posterior parietal cortex. From this important node, the dorsal pathway reaches the prefrontal, premotor, and medial temporal cortex (Kravitz et al., 2011). The parieto–prefrontal cortex is implicated in spatial working memory while the premotor cortex supports visually-guided action and the medial temporal cortex is implicated in navigation. The distribution on the scalp of the present N2-P3 ERP and the presence of theta ERS may indicate the contribution of the visual dorsal pathway during the PWL stimulation.

Because the present PWL is composed of different colors ranging from purple (415 nm) to red (720 nm), the activation of the short-wavelength (S) and long medium-wavelength (L/M) cones may logically contribute to some extent to the evoked response by the way of the parvocellular ventral pathway (Horwitz, 2020). Besides, it has been also proposed that some color signaling may reach the MT area of the dorsal pathway assuming a functional grouping of the parvocellular and magnocellular inputs (Conway, 2014).

It is important to note that the mixing nature of this PWL stimulation, including different colors and luminance motion, provides a definitive conclusion about a comparative analysis of the classical N2 related to the onset motion of simple visual item peaking at about 200 ms (Hirai et al., 2009) with the present N2. However, the longer latency of the present N2 (331 ms) could correspond to the second negativity related to the biological motion described by Krakowski et al. (2011) presenting a latency between 200 and 350 ms and a source bilaterally located in the posterior temporal cortex within the human homolog of the MT gyrus in monkeys and the posterior superior temporal sulcus. In the same trend, our N2 activity approximatively corresponds to the second component described by Hirai et al. (2003), the N2c of Inuggi et al. (2018), and “N300” named by Jokisch et al. (2005). Using coherent moving dots, Lange-Malecki et al. (2018) have also recorded similar fronto-central negativity (at FCz), peaking around 200–400 ms after the onset of the motion stimulus and called this component N2. Previously, we have shown that a similar ERSP during a video of human walking 3D-animation was also characterized by a delta–theta ERS of long duration accompanied by an alpha ERD (Zarka et al., 2014). Decreases in alpha power have been related to anticipative modulation of attention (Capotosto et al., 2009, 2012), visual perception, and encoding (Foxe and Snyder, 2011; Van Diepen et al., 2019). A significant ERD of the mu rhythms (8–13 Hz) in response to the biological motion was also described by Ulloa and Pineda (2007). After the N331 component, the P476 may correspond to the positivity recorded at centro-parietal electrodes starting after about 400 ms of biological motion when it was attended (Krakowski et al., 2011) and interpreted by these authors as associated with the deciphering of the meaning of the motion stimulus.

Although the present PWL motion was entirely different from biological motion, it could be possible that this late positivity represents a perceptual closure task (Krakowski et al., 2011) assumed by a top-down mechanism (Kröger et al., 2014) integrating a global moving wave effect produced by colored and luminance associated to breathing exercise. According to “embodied cognition” principles, the present active *RB* system associating PWL stimulation alternating eccentric and concentric motion with breathing maneuvers probably induces interoceptive signals about bodily states, which can modify the significance of the visual inputs (Belli et al., 2021) contributing to a peculiar abstract shaping of the brain states during the *RB* sessions.

The pulsed light effect mimicking concentric and eccentric waves may induce steady-state visual evoked potential-like (SSVEP-like) response and the possible entrainment of alpha neural generators during the *RB* sessions. However, the presence of numerous variations of color, luminance, and PWL frequency during the same session precluded the possibility to characterize such SSVEP-like in our participants. The possibility of the existence of a direct post-SSVEP effect during the AR testing must be taken into account. However, the recent study of Otero et al. (2020) demonstrated that the duration of the persistence effect of sinusoidally varying light stimulation inducing the SSVEP response was 241.30 ± 119.96 ms, which was too short for inducing a significant interference in the alpha power values obtained during the repeated AR measurements. In addition, the relatively low ITC values peaking at the same latency of the theta–alpha ERS could be explained by the fact that the entrainment effect was disturbed by the averaging of the different PWL frequencies.

### The Effects of Rebalance Session on the Alpha and Theta Oscillation

The *RB session* was able to significantly increase the power of alpha and the theta oscillations during the AR testing in all 14 subjects. Seven subjects have increased the alpha and theta power, three subjects only the theta power (they were alpha “non-responders”), and four subjects only the alpha power. We also found that a lower alpha power preceding the *RB* session was linked to an effective increase of the alpha power during the *RB* session in the EC state. Neither the PIR nor the POR control groups show any significant variation.

Even though the alpha oscillation (peaking around 10 Hz) is the dominant EEG rhythm in awake relaxed subjects, their basic physiological understanding and related functions remind largely open to discussion (Cheron et al., 2006, 2016; Foxe and Snyder, 2011; Sadaghiani and Kleinschmidt, 2016; Cebolla and Cheron, 2019; Van Diepen et al., 2019; Samaha et al., 2020; Wilson and Foxe, 2020). In an earlier reported by Berger (1929), the dominant alpha rhythm significantly increases when the eyes are closed and decreases when the eyes are opened, which is called the AR achieved in numerous studies (Adrian and Matthews, 1934; Klimesch et al., 1997; Klimesch, 1999; Cheron et al., 2006; Bazanova and Vernon, 2014). Interestingly, alpha can also increase when the eyes are open if an opal glass bowl is placed in front of the participant’s face producing a uniform visual field (Figure 9 of Adrian and Matthews, 1934) and once it reaches a certain level it potentially remain increased even if the illumination of the bowl is modified Adrian and Matthews (1934).

In our study, the alpha oscillation in the EO was performed in darkness in the passive *RB* environment, which might help the alpha occurrence. This corroborates more recent studies showing that alpha oscillation with eyes open was reinforced in a dark quiet room during the multimodal Ganzfeld, which consisted of an audio-visual environment with an unfilled homogenous color visual field and waterfall noise (Pütz et al., 2006; Wackermann et al., 2008; Miskovic et al., 2019). A recent MEG study by Popov et al. (2019) demonstrated that the alpha power modulation of the visual cortex is retinotopically organized and that alpha ERD reflects a gain increase or engagement in the attended visual field while alpha ERS reflects a gain decrease or disengagement in the unattended field. Popov et al. (2019) insisted that these modulations are due to internal attention rather than an external stimulus input. We did not find such hemispherical alpha modulation in our study which could be explained by the type of visual stimulation used here which covered the entire visual field and the fact that there was not any spatial attentional demand. Considering that alpha showed ERD during the *RB* sessions, we speculate that the alpha power increase during the AR testing could be due to a rebound effect as is explained by the inhibition hypothesis of Klimesch et al. (2007).

It has been reported that the alpha oscillation power increase on the right frontal area is correlated with spiritual experience during a sensory deprivation environment (Glicksohn and Ben-Soussan, 2020). We did not find the alpha but theta power increase in frontal regions; no participant of our study reported any spiritual or mystic experience during the *RB* sessions.

### The Individual Alpha Peak Frequency Was Not Modified by Rebalance Sessions

Participants’ mean individual alpha peak frequency (IAPF) corresponds to the higher IAPF values reported by Bazanova and Vernon (2014) in 96 healthy subjects, which could be explained by the fact that the majority of our participants belonged to sports elites and that the IAPF was known to be higher in good performers [cognitive composite performance (Bazanova et al., 2009; Grandy et al., 2013a,b)]. On the contrary, IAPF decrease is related to the occurrence of fatigue, bad performance (Klimesch et al., 1993), and cognitive preparedness (Grandy et al., 2013a,b). The IAPF remained stable in our study.

Theta rhythm is considered as a basic physiological element of the global oscillatory synchronization processes connecting multiple brain regions (Buzsáki and Draguhn, 2004; Buzsáki, 2005; Fries, 2005). A clear theta peak at ∼6 Hz has been recorded in the human hippocampus (Kahana et al., 1999) during the navigation task (Kaplan and Friston, 2018). Interestingly, the duration of the theta ERS produced by the onset of navigational movement was about 300 ms, a value very close to the present ERS evoked by the PWL onset. In human intracranial EEG recordings, Raghavachari et al. (2006) have also reported the theta oscillation during a working memory task in the middle frontal gyrus. In addition, the theta oscillation is in close connection with the activities of the DMN (Raichle et al., 2001; Hsieh and Ranganath, 2014; Raichle, 2015), and the theta in the frontal midline (FMT) is negatively correlated with blood oxygenation level-dependent (BOLD) signal of the DMN (Scheeringa et al., 2008). As the same correlation was reported between a high power of alpha oscillation and a low BOLD signal, we may speculate that the *RB* session could favor a cerebral low energetic demand.

The multiplicity of functional roles played by this oscillation concern also the memory function. It has been demonstrated that the theta power increase occurred during successful encoding and promoted the emergence of gamma oscillation being linked to the formation of new episodic memories (Sederberg et al., 2003). Still, the relation between memory formation and theta oscillation is not simple and it depends on context matching. Staudigl and Hanslmayr (2013) demonstrated that high theta power increased when the memory encoding was associated with a successful recognition, whereas it decreased in the opposite case. During working memory retrieval, the number of to-be-distinguished items is correlated with an increase of the theta power (Meyer et al., 2015). The theta oscillation contributes to setting the dynamics of memory encoding and retrieval inside the cortical circuits (Hasselmo and Stern, 2014) engaging the spatio-temporal coding realized into the hippocampus and entorhinal cortex. In addition and considering that spatial attention is a discontinuous process, intrinsic frontoparietal theta, assumes such rhythmic sampling formed by two attentional states alternating between engagement (enhanced perception) and disengagement (reduced perception) (Fiebelkorn and Kastner, 2018, 2020). It was demonstrated in the macaque that these theta-dependent states are coordinated by the pulvinar nucleus (Fiebelkorn et al., 2019).

We may speculate that in the present situation, the PWL induced a visual stimulus-driven condition without a visual goal-directed condition while the breathing demands created a mixing situation where the theta could play an integrative rule.

The modulation of ongoing theta oscillations implicating long-range theta coherence across the neocortex has also been found during the decision process (Cohen and Donner, 2013). In this context, it has been recently proposed that theta coherence between the medial prefrontal cortex (mPFC) and the anterior cingulate cortex (ACC) predicts the speed and the task performance and represents a crucial mechanism for a performing cognitive control (Myers et al., 2021). In the same way, numerous studies (Helfrich et al., 2019; Kaiser and Schütz-Bosbach, 2021) have suggested that the theta oscillation assumed a general control mechanism, exerting a top-down control during both sensorimotor interference and goal behavior. Gärtner et al. (2014) demonstrated the existence of a close link between working memory performance and the theta oscillation (4–8 Hz) which were both reduced under stress conditions. Furthermore, this FM theta helps to maintain the working memory process and is significantly vulnerable to aging (Tóth et al., 2014).

A recent study dedicated to the detection of Alzheimer’s disease using EEG (Özbek et al., 2021) showed that alpha and theta powers were higher in young than in the older healthy subject. All these might suggest that the oscillatory power increases observed after the *RB* session might reflect a positive outcome.

The theta and alpha oscillations shared similar properties (Bruegger and Abegg, 2021; Liang et al., 2021). For example, their frequency and power have been linked to attention and perception. The fact that the power of both oscillations was significantly increased but with the maintenance of their frequency peak during the *RB* session points to a common gain effect exerted by the combination of PWL and breathing exercises. For Aftanas and Golocheikine (2001), the selective increase of theta and alpha oscillating contribution during meditative practice may be viewed with the states of internalized attention and emotionally positive “blissful” experience as those often reported by the practitioners of *RB* devices.

Despite the heterogeneity of meditative methods (Deolindo et al., 2020), the enhancement of the theta oscillation (Tanaka et al., 2014; DeLosAngeles et al., 2016; Tang et al., 2019) and its synchronization throughout multiple areas of the brain (Wong et al., 2015; Lardone et al., 2018) represent consistent results, which might be linked to the power theta increase reproduced in this study. Because PWL stimulation and breathing exercises are potentially able to induce oscillatory modulation of the brain rhythms, it is at first sight difficult to dissociate in the present effects those due to guided breathing from those due to the PWL stimulation. The absence of breathing during S2, S5, and S6 indicates that breathing was not the raison of the alpha and theta power behavior, which was maintained at higher levels during AR3, AR6, and AR7. This suggests that the effect of the PWL chromotherapy alone (in the absence of the guided breathing) was able to induce theta–alpha oscillation potentiation.

It is important to note that the present results cannot dissociate the effects of the stimulation from the athletic level on the participants and that other non-stimulation-related factors might have contributed to the observed group differences. Further studies will need to integrate control groups composed of athletes distributed in AIR, PIR, and POR conditions. Based on these preliminary results, we may advance that the present chromotherapy PWL stimulation and guided relaxation, inducing oscillatory entrainment can rapidly increase the power of theta–alpha brain oscillations measured during EC–EO wakefulness states. This could be further explored as complementary neuropsychiatric perspectives.

## Data Availability Statement

The raw data supporting the conclusions of this article will be made available by the authors, without undue reservation.

## Ethics Statement

The studies involving human participants were reviewed and approved by Ethics committee of Université Libre de Bruxelles, CHU Brugmann, and conducted in conformity with the European Union directive 2001/20/EC of the European Parliament. The patients/participants provided their written informed consent to participate in this study.

## Author Contributions

A-MC and GC conceived the original idea. GC, MP, and CS designed the experiment. MP, DR, A-MC, DZ, and GC performed the experiments. DR and MP performed the data analysis. GC wrote the manuscript. A-MC contributed to the writing of this manuscript. All authors contributed to the article and approved the submitted version.

## Conflict of Interest

The authors declare that the research was conducted in the absence of any commercial or financial relationships that could be construed as a potential conflict of interest.

## Publisher’s Note

All claims expressed in this article are solely those of the authors and do not necessarily represent those of their affiliated organizations, or those of the publisher, the editors and the reviewers. Any product that may be evaluated in this article, or claim that may be made by its manufacturer, is not guaranteed or endorsed by the publisher.

## Footnotes

1
https://rebalance-impulse.com/
