# Supplementary material for: Effects of Pulsed-Wave Chromotherapy and Guided Relaxation on the Theta-Alpha Oscillation During Arrest Reaction
Source: Front Psychol. 2022 Mar 3;13:792872. doi: 10.3389/fpsyg.2022.792872 (PMC8929400; doi:10.3389/fpsyg.2022.792872)
Supplement: Supplementary file 1 [file Presentation_1.PPTX]

## Slide 1
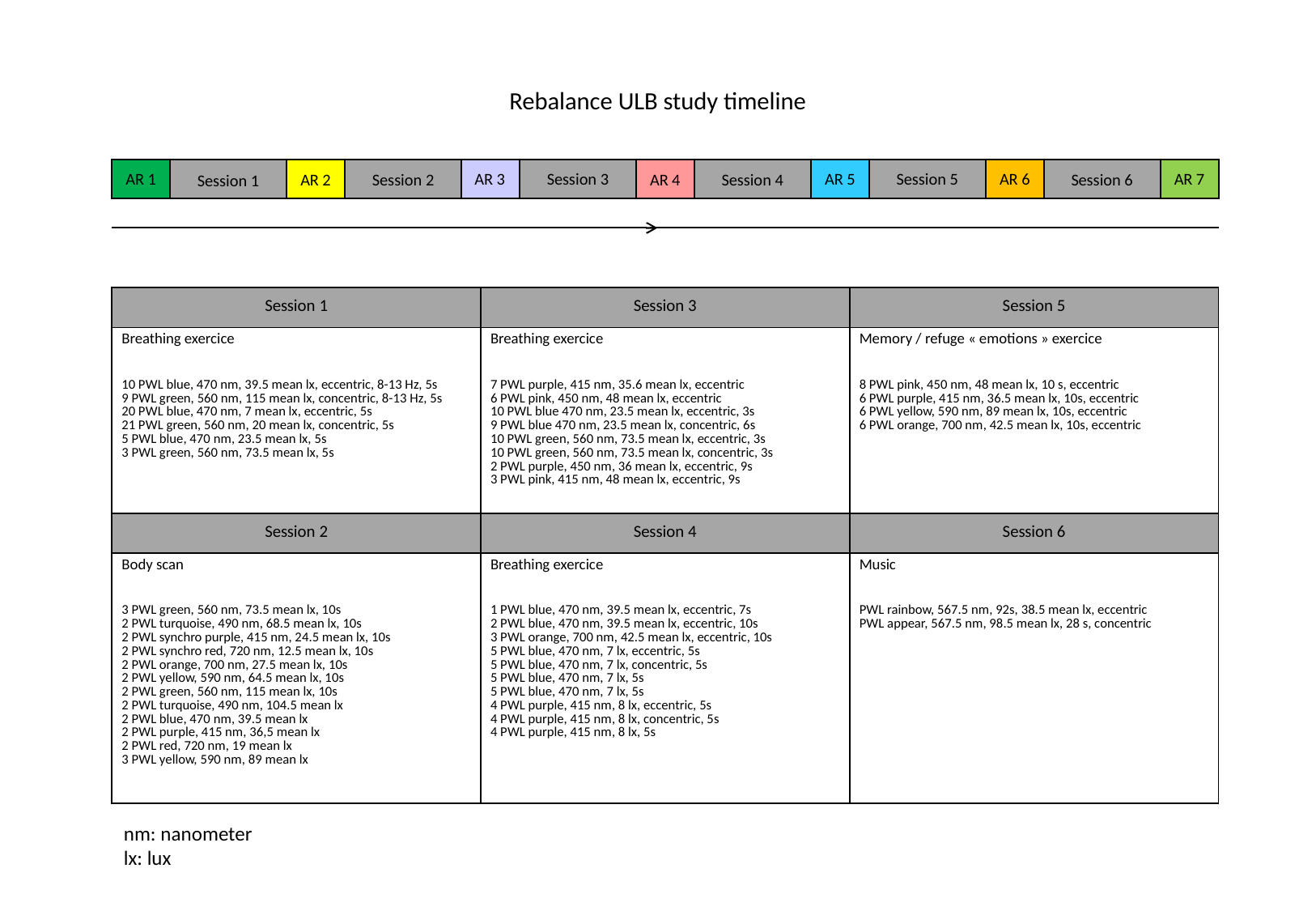

Rebalance ULB study timeline
AR 1
AR 3
Session 5
AR 7
Session 3
AR 5
AR 6
AR 4
Session 6
Session 4
AR 2
Session 2
Session 1
| Session 1 | Session 3 | Session 5 |
| --- | --- | --- |
| Breathing exercice | Breathing exercice | Memory / refuge « emotions » exercice |
| 10 PWL blue, 470 nm, 39.5 mean lx, eccentric, 8-13 Hz, 5s 9 PWL green, 560 nm, 115 mean lx, concentric, 8-13 Hz, 5s 20 PWL blue, 470 nm, 7 mean lx, eccentric, 5s 21 PWL green, 560 nm, 20 mean lx, concentric, 5s 5 PWL blue, 470 nm, 23.5 mean lx, 5s 3 PWL green, 560 nm, 73.5 mean lx, 5s | 7 PWL purple, 415 nm, 35.6 mean lx, eccentric 6 PWL pink, 450 nm, 48 mean lx, eccentric 10 PWL blue 470 nm, 23.5 mean lx, eccentric, 3s 9 PWL blue 470 nm, 23.5 mean lx, concentric, 6s 10 PWL green, 560 nm, 73.5 mean lx, eccentric, 3s 10 PWL green, 560 nm, 73.5 mean lx, concentric, 3s 2 PWL purple, 450 nm, 36 mean lx, eccentric, 9s 3 PWL pink, 415 nm, 48 mean lx, eccentric, 9s | 8 PWL pink, 450 nm, 48 mean lx, 10 s, eccentric 6 PWL purple, 415 nm, 36.5 mean lx, 10s, eccentric 6 PWL yellow, 590 nm, 89 mean lx, 10s, eccentric 6 PWL orange, 700 nm, 42.5 mean lx, 10s, eccentric |
| Session 2 | Session 4 | Session 6 |
| Body scan | Breathing exercice | Music |
| 3 PWL green, 560 nm, 73.5 mean lx, 10s 2 PWL turquoise, 490 nm, 68.5 mean lx, 10s 2 PWL synchro purple, 415 nm, 24.5 mean lx, 10s 2 PWL synchro red, 720 nm, 12.5 mean lx, 10s 2 PWL orange, 700 nm, 27.5 mean lx, 10s 2 PWL yellow, 590 nm, 64.5 mean lx, 10s 2 PWL green, 560 nm, 115 mean lx, 10s 2 PWL turquoise, 490 nm, 104.5 mean lx 2 PWL blue, 470 nm, 39.5 mean lx 2 PWL purple, 415 nm, 36,5 mean lx 2 PWL red, 720 nm, 19 mean lx 3 PWL yellow, 590 nm, 89 mean lx | 1 PWL blue, 470 nm, 39.5 mean lx, eccentric, 7s 2 PWL blue, 470 nm, 39.5 mean lx, eccentric, 10s 3 PWL orange, 700 nm, 42.5 mean lx, eccentric, 10s 5 PWL blue, 470 nm, 7 lx, eccentric, 5s 5 PWL blue, 470 nm, 7 lx, concentric, 5s 5 PWL blue, 470 nm, 7 lx, 5s 5 PWL blue, 470 nm, 7 lx, 5s 4 PWL purple, 415 nm, 8 lx, eccentric, 5s 4 PWL purple, 415 nm, 8 lx, concentric, 5s 4 PWL purple, 415 nm, 8 lx, 5s | PWL rainbow, 567.5 nm, 92s, 38.5 mean lx, eccentric PWL appear, 567.5 nm, 98.5 mean lx, 28 s, concentric |
nm: nanometer
lx: lux

## Slide 2
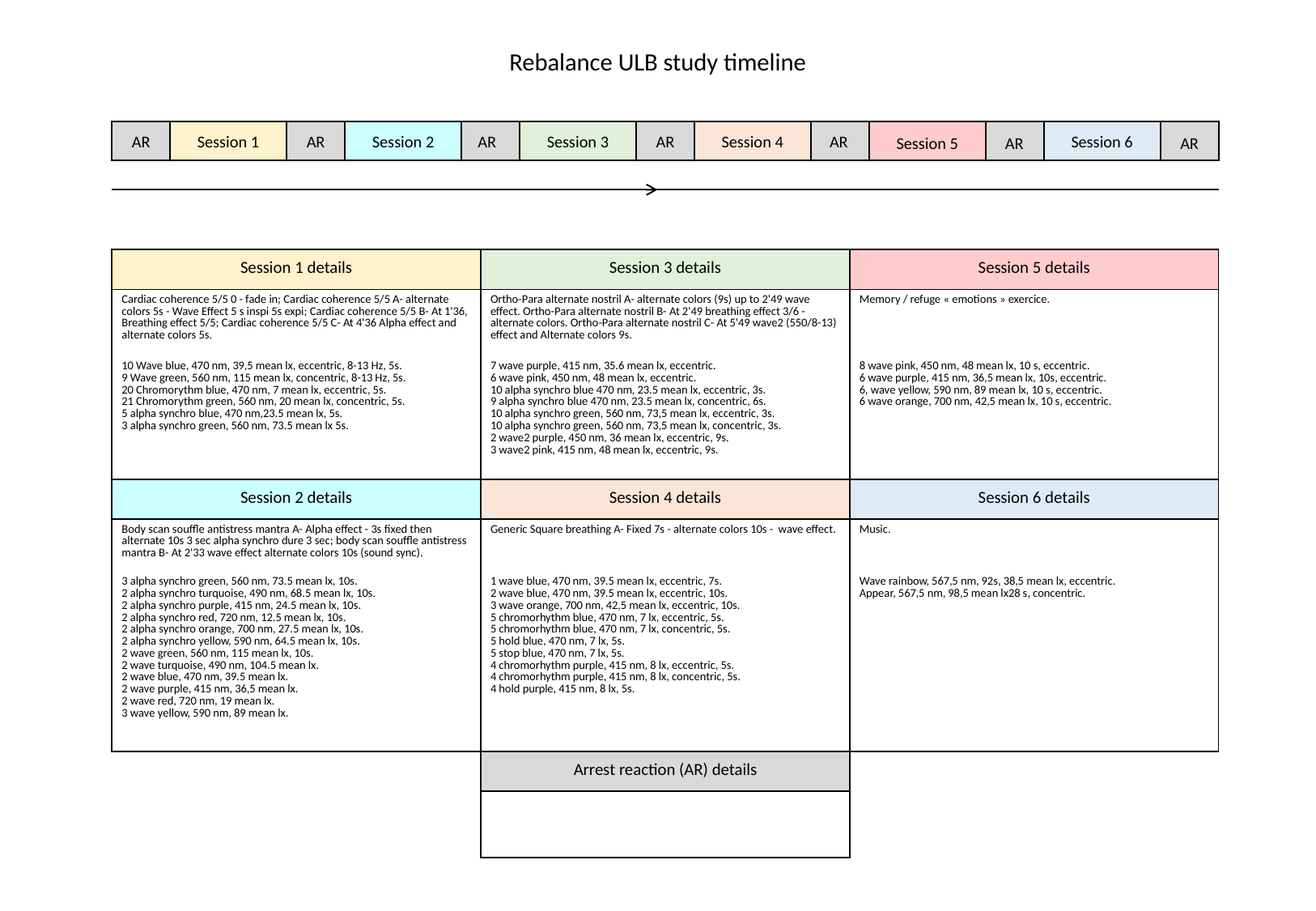

Rebalance ULB study timeline
AR
AR
AR
AR
AR
Session 6
Session 4
Session 1
Session 2
Session 3
AR
Session 5
AR
| Session 1 details | Session 3 details | Session 5 details |
| --- | --- | --- |
| Cardiac coherence 5/5 0 - fade in; Cardiac coherence 5/5 A- alternate colors 5s - Wave Effect 5 s inspi 5s expi; Cardiac coherence 5/5 B- At 1'36, Breathing effect 5/5; Cardiac coherence 5/5 C- At 4'36 Alpha effect and alternate colors 5s. | Ortho-Para alternate nostril A- alternate colors (9s) up to 2'49 wave effect. Ortho-Para alternate nostril B- At 2'49 breathing effect 3/6 - alternate colors. Ortho-Para alternate nostril C- At 5'49 wave2 (550/8-13) effect and Alternate colors 9s. | Memory / refuge « emotions » exercice. |
| 10 Wave blue, 470 nm, 39,5 mean lx, eccentric, 8-13 Hz, 5s. 9 Wave green, 560 nm, 115 mean lx, concentric, 8-13 Hz, 5s. 20 Chromorythm blue, 470 nm, 7 mean lx, eccentric, 5s. 21 Chromorythm green, 560 nm, 20 mean lx, concentric, 5s. 5 alpha synchro blue, 470 nm,23.5 mean lx, 5s. 3 alpha synchro green, 560 nm, 73.5 mean lx 5s. | 7 wave purple, 415 nm, 35.6 mean lx, eccentric. 6 wave pink, 450 nm, 48 mean lx, eccentric. 10 alpha synchro blue 470 nm, 23.5 mean lx, eccentric, 3s. 9 alpha synchro blue 470 nm, 23.5 mean lx, concentric, 6s. 10 alpha synchro green, 560 nm, 73,5 mean lx, eccentric, 3s. 10 alpha synchro green, 560 nm, 73,5 mean lx, concentric, 3s. 2 wave2 purple, 450 nm, 36 mean lx, eccentric, 9s. 3 wave2 pink, 415 nm, 48 mean lx, eccentric, 9s. | 8 wave pink, 450 nm, 48 mean lx, 10 s, eccentric. 6 wave purple, 415 nm, 36,5 mean lx, 10s, eccentric. 6, wave yellow, 590 nm, 89 mean lx, 10 s, eccentric. 6 wave orange, 700 nm, 42,5 mean lx, 10 s, eccentric. |
| Session 2 details | Session 4 details | Session 6 details |
| Body scan souffle antistress mantra A- Alpha effect - 3s fixed then alternate 10s 3 sec alpha synchro dure 3 sec; body scan souffle antistress mantra B- At 2'33 wave effect alternate colors 10s (sound sync). | Generic Square breathing A- Fixed 7s - alternate colors 10s - wave effect. | Music. |
| 3 alpha synchro green, 560 nm, 73.5 mean lx, 10s. 2 alpha synchro turquoise, 490 nm, 68.5 mean lx, 10s. 2 alpha synchro purple, 415 nm, 24.5 mean lx, 10s. 2 alpha synchro red, 720 nm, 12.5 mean lx, 10s. 2 alpha synchro orange, 700 nm, 27.5 mean lx, 10s. 2 alpha synchro yellow, 590 nm, 64.5 mean lx, 10s. 2 wave green, 560 nm, 115 mean lx, 10s. 2 wave turquoise, 490 nm, 104.5 mean lx. 2 wave blue, 470 nm, 39.5 mean lx. 2 wave purple, 415 nm, 36,5 mean lx. 2 wave red, 720 nm, 19 mean lx. 3 wave yellow, 590 nm, 89 mean lx. | 1 wave blue, 470 nm, 39.5 mean lx, eccentric, 7s. 2 wave blue, 470 nm, 39.5 mean lx, eccentric, 10s. 3 wave orange, 700 nm, 42,5 mean lx, eccentric, 10s. 5 chromorhythm blue, 470 nm, 7 lx, eccentric, 5s. 5 chromorhythm blue, 470 nm, 7 lx, concentric, 5s. 5 hold blue, 470 nm, 7 lx, 5s. 5 stop blue, 470 nm, 7 lx, 5s. 4 chromorhythm purple, 415 nm, 8 lx, eccentric, 5s. 4 chromorhythm purple, 415 nm, 8 lx, concentric, 5s. 4 hold purple, 415 nm, 8 lx, 5s. | Wave rainbow, 567,5 nm, 92s, 38,5 mean lx, eccentric. Appear, 567,5 nm, 98,5 mean lx28 s, concentric. |
| | Arrest reaction (AR) details | |
| | | |
